# Taxonomic review of genus *Microrhagus* Dejean, 1833 from Korea, with description of a new species (Coleoptera, Eucnemidae, Melasinae, Dirhagini)

**DOI:** 10.3897/zookeys.781.21106

**Published:** 2018-08-13

**Authors:** Jinbae Seung, Seunghwan Lee

**Affiliations:** 1 Insect Biosystematics Laboratory, Department of Agricultural Biotechnology, Seoul National University, Seoul 151-921, Republic of Korea Seoul National University Seoul Korea, South; 2 Research Institute for Agricultural and Life Sciences, Seoul National University, Seoul 151-921, Republic of Korea Seoul National University Seoul Korea, South

**Keywords:** Eucnemidae, Korea, *
Microrhagus
*, new species, taxonomy

## Abstract

The genus *Microrhagus* Dejean, 1833 is reviewed with four species from Korea: *Microrhagusfoveolatus* (Fleutiaux, 1923), *Microrhagusjejuensis***sp. n.**, *Microrhagusmystagogus* (Fleutiaux, 1923), and *Microrhagusramosus* (Fleutiaux, 1902). Herein, all Korean *Microrhagus* species are redescribed. A key to species of Korean *Microrhagus* and photographs of the diagnostic characters are also provided.

## Introduction

The tribe Dirhagini Reitter, 1911 is a large group of the subfamily Melasinae with a worldwide distribution. Dirhagini is characterized by crenulate or incomplete pronotal lateral carina, most also with well-developed notosternal antennal grooves, an apical sex-comb on male protarsomere I, and without basal struts of the aedeagal median lobe (Muona 1993, 2011).

The genus *Microrhagus* Dejean, 1833 is the largest genus in its tribe, with more than 130 species worldwide and 17 species in the Palaearctic Region, including seven species from Japan (Muona 2007, 2011; Kovalev 2013, 2016). Only *Microrhagusramosus* was known from Korea (Suzuki 2014).

The genus *Microrhagus* is distinguished from other genera by strongly approximate antennal sockets, hypomeron with pits close to procoxae, well-developed notosternal antennal grooves, simple elytral apices, laterally narrowed metacoxal plates, and apically notched median lobe and long ventral lobe of aedeagus (Fleutiaux 1935; Hisamatsu 1960; Muona 2000).

We review genus *Microrhagus* with four species, including an undescribed species, *Microrhagusjejuensis* sp. n., and two previously unreported species, *Microrhagusfoveolatus* (Fleutiaux, 1923) and *M.mystagogus* (Fleutiaux, 1923) from Korea. A key to species of Korean *Microrhagus*, diagnoses, redescriptions, and photographs for the diagnostic characters are provided.

## Materials and methods

Most samples examined were collected using flight intercept traps between 2015 and 2016. Samples were preserved in 95% ethanol and made into dried specimens by double mounted method (pinned with a micropin to a block of cork, which is mounted on a standard insect pin) for exact identification. To examine the antennae, legs, and aedeagus, specimens were softened in boiling water for 30–60 minutes and dissected using forceps and micro-pin probes. Dried specimens were examined under a microscope (S8APO, Leica, Germany) and separate organs were observed under a microscope (DM4000B, Leica, Germany). Photographs were taken using EOS-600D, CANON camera, through MP-E 65mm lens.

All specimens (including types) are deposited in the insect collection of the College for Agriculture and Life Sciences, Seoul National University (CALS, SNU, Seoul, Korea).

The concept of genus *Microrhagus* follows Muona (1993) and morphological terminology follows Muona (1993) and Otto (2016). Lateral lobes and secondary lateral lobes of aedeagus refer to the lateral and mesal apices of the parameres, respectively.

Identification of species was done using Fleutiaux (1902, 1923, 1935) and Hisamatsu (1960, 1985). Identifications were rechecked by J. Muona who has examined the relevant type specimens of Palaearctic species of *Microrhagus*, including *Microrhaguspectinicornis*, closely similar to new species. Also, W. Suzuki confirmed identifications in process.

## Results

### Family Eucnemidae Eschscholtz, 1829

#### Subfamily Melasinae Fleming, 1821

##### Tribe Dirhagini Reitter, 1911

###### Genus Microrhagus

Taxon classificationAnimaliaColeopteraEucnemidae

Dejean, 1833

Microrhagus Dejean, 1833: 85. Type species: Elaterpygmaeus Fabricius, 1792.

####### Diagnosis.

Head: pits present between eyes and antennal sockets; frontoclypeal region strongly narrowed at base; antennae serrate or pectinate; antennomere III not longer than antennomeres IV–V combined. Prothorax: pronotal lateral carina divided into anterolateral carina and posterolateral carina; notosternal antennal grooves well developed. Pterothorax: mesepimeron fused with mesepisternum; metepisternum subparallel-sided or widened posteriorly; metacoxal plate expanded inward. Leg: male protarsomere I with apical sex-comb; metatarsomere I not shorter than metatarsomeres II–III combined, metatarsomere IV slightly dilated. Abdomen: abdominal ventrites connate; abdominal ventrite V obtusely produced or simply rounded at apex in ventral view; median lobe of aedeagus bifurcate at apex and secondary lateral lobes small (de Bonvouloir 1872; Reitter 1921; Fleutiaux 1923, 1935; Hisamatsu 1960, 1985; Muona 2000).

######## Key to species of Korean *Microrhagus*

**Table:** 

1	Frons with carina or groove at midline; pronotum narrowed anteriorly, with paired dimples near middle; male antennae pectinate from antennomere III	**2**
–	Frons without carina or groove (Fig. 3G); pronotum nearly parallel-sided, without dimples; male antennae pectinate from antennomere IV (Fig. 3E)	***M.mystagogus* (Fleutiaux)**
2	Pronotum without groove at midline; elytra less than 2.5 × longer than combined width; male antennomere III with process at base, antennomere IV with process at mid-length, antennomeres V–X with processes at apex; lateral lobes of aedeagus narrowly produced apically	**3**
–	Pronotum with a longitudinal groove at midline (Fig. 2A, B); elytra 2.7 × longer than combined width; male antennomeres III–X with process near apex (Fig. 2E); lateral lobes of aedeagus truncate (Fig. 2M)	***M.jejuensis* sp. n.**
3	Pronotum less densely punctate, average distance between punctures greater than puncture diameter; antennal process III as long as length of antennomere III (Fig. 1E); ventral lobe of aedeagus broadened toward apex (Fig. 1M)	***M.foveolatus* (Fleutiaux)**
–	Pronotum very densely punctate, average distance between punctures smaller than puncture diameter; antennal process III 1.4 × longer than length of antennomere III (Fig. 4E); ventral lobe of aedeagus subparallel-sided (Fig. 4M)	***M.ramosus* Fleutiaux**

###### Microrhagus foveolatus

Taxon classificationAnimaliaColeopteraEucnemidae

(Fleutiaux, 1923)

Fig. 1

Dirhagus foveolatus Fleutiaux, 1923: 308.

####### Diagnosis.

Body: mostly shiny black. Head: frons with a weak carina at midline; antennae pectinate from antenomere III in male. Prothorax: pronotum with sparse punctures, average distance between punctures greater than puncture diameter, disc with paired dimples near middle; notosternal antennal grooves slightly widened posteriorly. Pterothorax: elytra 2.5 × longer than combined width; metepisternum gradually widened posteriorly, its greatest width narrower than outer edge of metacoxal plate; metacoxal plate expanded inward; abdominal ventrite V narrowly rounded at apex.

####### Redescription.

**Figure 1.**
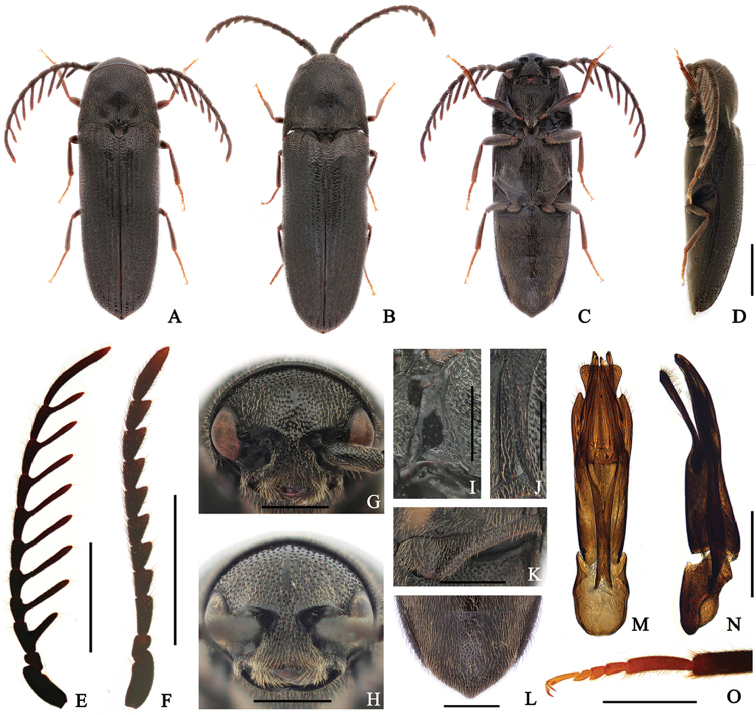
*Microrhagusfoveolatus* (Fleutiaux, 1923). **A, C–E, G, I–O** male **B, F, H** female. **A, B** dorsal habitus **C** ventral habitus **D** lateral habitus **E–F** antenna **G–H** frons **I** antennal groove **J** metepisternum **K** metacoxal plate **L** abdominal ventrite V **M–N** aedeagus **O** metatarsus. Scale bar: 1 mm (**A–F**); 0.5 mm (**G–O**).

**Male** (Fig. 1A, C–D) 5.1–6.0 mm long and 1.6–1.9 mm wide. **Body** mostly black; tarsi yellow-brown; surface glossy, with yellow pubescence. **Head** with circular and regularly sized punctures, denser at frontoclypeal region; frons with a weak carina at midline; frontoclypeal region slightly depressed at base, broadly rounded, with anterior edge slightly sinuate, anterior edge 3.7 × wider than distance between antennal sockets (Fig. 1G). **Antennae** (Fig. 1E) almost reaching metacoxal plate, with yellow-brown pubescence, and pectinate from antennomere III; processes of antennomeres III, IV, and V 1.1, 2.1 and 2.1 × as long as corresponding antennomeres; antennomere I robust; antennomere II shortest; antennomere III with process near base, 1.7 × longer than II, and 1.3 × longer than IV; antennomere IV with process at mid-length; antennomeres V–X with processes near apex; apical antennomere strongly elongate, curved, 9.5 × longer than wide, and 2.5 × longer than previous one. **Pronotum** 1.2 × wider than long, subparallel-sided near base, gradually narrowed anteriorly; surface with punctures, average distance between punctures greater than puncture diameter; disc with paired dimples near middle and a short carina at base of midline; anterolateral carina exceeding half as long as pronotum; posterolateral carina almost reaching pronotal mid-length, fused with anterolateral carina in some; antescutellar area almost straight in dorsal view; pronotal posterior angles sharply projecting, exceeding posterior edge of antescutellar area. **Scutellum** slightly raised, 1.1 × longer than wide, gradually narrowed posteriorly, and rounded at apex; surface coarse, densely punctate, pubescent with dense setae, especially at apex. **Elytra** 2.5 × longer than combined width, gradually narrowing posteriorly; disc striate, with irregularly sized and spaced punctures; interstriae moderately convex, with several large, deep punctures near apices; apices simply rounded. **Prosternum** with curved sides, anterior margin shallowly bisinuate; punctures slightly denser anteriorly and posteriorly; prosternal process stout, tapered and curved dorsally at posterior end; hypomeron with punctures less than on prosternum; notosternal antennal grooves (Fig. 1I) slightly expanded posteriorly, sparsely punctate, glabrous, and with pits. **Mesoventrite** with irregularly sized punctures; mesopleuron with rough surface, especially anteriorly. **Metaventrite** with finer and denser punctures than on prosternum, especially at middle; disc with a weak median groove, not reaching anterior edge; metepisternum (Fig. 1J) gradually widened posteriorly, its greatest width four-fifths of outer edge of metacoxal plate; metacoxal plate (Fig. 1K) expanded inward, medially 2.3 × wider than laterally. **Legs** (Fig. 1O) slender; metatarsomere I 1.2 × longer than II–IV combined; metatarsomere II 1.3 × longer than III; metatarsomere V 1.7 × longer than II; claws simple. **Abdomen** with finer punctures than metaventrite; ventrite V narrowly rounded at apex (Fig. 1L). **Aedeagus** (Fig. 1M–N) 4.3 × longer than wide; median lobe slightly curved ventrally, bifurcate at apex; lateral lobes as long as median lobe, feebly curved ventrally, blunt at apex, and with basally attached secondary lateral lobes; secondary lateral lobes shorter than lateral lobes, bent ventrally, parallel-sided, blunt at apex, and with long setae; ventral lobe as long as median lobe, gradually expanded apically, broadly truncate at apex, and densely pubescent; phallobase rectangular, 1.8 × longer than wide, one-third as long as entire aedeagus. **Female** (Fig. 1B) is distinguished from male by following characters: body stouter, 5.3–6.4 mm long and 1.7–2.1 mm wide; frontoclypeal region with anterior edge, 3.5 × wider than distance between antennal sockets (Fig. 1H); antennae (Fig. 1F) serrate, not reaching metacoxal plate; antennomere II short, as long as IV; antennomere III 1.9 × longer than wide, 1.7 × longer than each length of II and IV; antennomeres IV–X gradually increasing in length, narrowing, and more strongly toothed toward antennal apex; apical antennomere 3.5 × longer than wide, 2.2 × longer than X.

####### Specimens examined.

**<Seoul>** 1♀, Gil-dong Natural Ecology Park, Gildong, Gangdong-gu, Seoul-si, N37°32'31.56", E127°9'18.58", 56m alt., 16 May, 2016, B. H. Jung leg. (SNU). **<Gyeonggi-do>** 1♀, Deoksu-ri, Danwol-myeon, Yangpyeong-gun, N37°33'22.97", E127°41'13.65", 166m alt., Flight intercept trap, 08–22 May, 2016, Seung and Jung leg. (SNU). **<Gangwond-do>** 1♀, Suha-ri, Daegwanryeong-myeon, Pyeongchang-gun, N37°36'36.29", E128°43'11.47", 803m alt., flight intercept trap, 05–29 June, 2016, Seung and Jung leg. (SNU); 1♀, Hoenggye-ri, Daegwanryeong-myeon, Pyeongchang-gun, N37°40'58.95", E128°45'21.80", 830m alt., flight intercept trap, 05–29 June, 2016, Seung and Jung leg. (SNU). **<Jeju Is.>** 1♂1♀, Gyorae gotjawal, Gyorae-ri, Jocheon-eup, Jeju-si, N33°26'21.88", E126°40'12.16", 422m alt. 10 June, 2016, J. B. Seung leg. (SNU); 2♂, Gyorae gotjawal, Gyorae-ri, Jocheon-eup, Jeju-si, N33°26'21.15", E126°40'12.75", 428m alt., flight intercept trap, 13 May–10 June, 2016, Seung and Jung leg. (SNU); 1♂, Seongpanak, Gyorae-ri, Jocheon-eup, Jeju-si, N33°23'10.82", E126°37'13.77", 752m alt., flight intercept trap, 13 May–10 June, 2016, Seung and Jung leg. (SNU).

####### Distribution.

Korea (New record), Japan, Russia (Far East).

####### Remarks.

*Microrhagusfoveolatus* is differentiated from *M.ramosus* by following characters: with relatively shorter antennal processes; pronotum with sparser punctures, distance between punctures greater than its diameter; elytra relatively more elongate; ventral lobe of aedeagus gradually broadened toward apex. Adults were observed on a standing dead tree with peeling loose bark and covered with hyphae.

###### Microrhagus jejuensis sp. n.

Taxon classificationAnimaliaColeopteraEucnemidae

http://zoobank.org/FDACE8CB-A261-4A41-B19F-15233F45707F

Fig. 2

####### Diagnosis.

Body: shiny black. Head: frons with a groove at midline; antennae pectinate from antennomere III in male. Prothorax: pronotum with paired dimples at middle and a groove at midline; notosternal antennal grooves slightly widened posteriorly, with outer marginal carina. Pterothorax: scutellum slightly elevated; elytra 2.7 × longer than combined width; metepisternum narrow, slightly widened posteriorly, its greatest width as wide as outer edge of metacoxal plate; metacoxal plate expanded inward. Abdomen: abdominal ventrite V narrowly rounded at apex.

####### Description.

**Figure 2.**
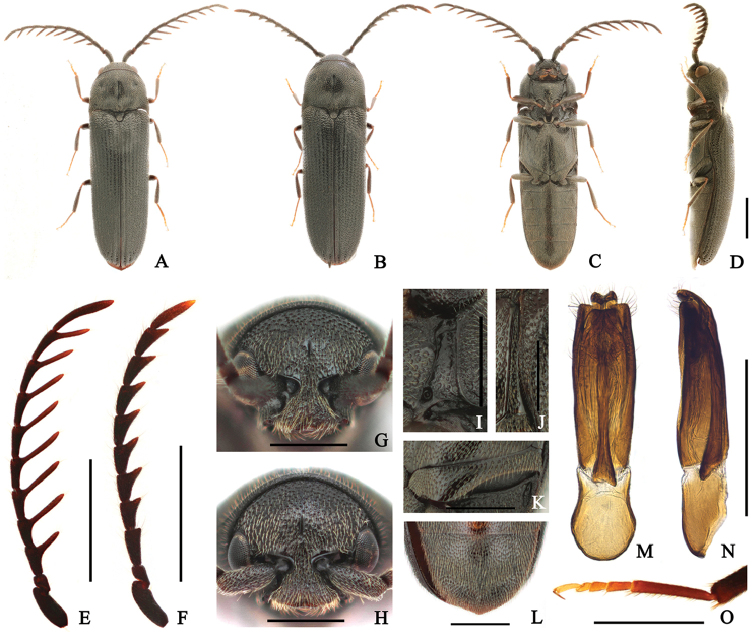
*Microrhagusjejuensis* sp. n. **A, C–E, G, I–O** male **B, F, H** female. **A, B**, dorsal habitus **C** ventral habitus **D** lateral habitus **E–F** antenna **G–H** frons **I** antennal groove **J** metepisternum **K** metacoxal plate **L** abdominal ventrite V **M–N** aedeagus **O** metatarsus. Scale bar: 1 mm (**A–F**); 0.5 mm (**G–O**).

**Holotype male** (Fig. 2A, C, D) 5.2 mm long and 1.5 mm wide. **Body** mostly black; antennomere II, antennal processes, mandible, and tibiae red-brown; maxillary palpi and tarsi yellow-brown; surface glossy, with yellow pubescence. **Head** with circular, irregularly sized punctures, especially at frontoclypeal region; frons with a short median groove; frontoclypeal region slightly depressed at base, broadly bifurcate and feebly concave at anterior edge, anterior edge 3.9 × wider than distance between antennal sockets (Fig. 2G). **Antennae** (Fig. 2E) almost exceeding abdominal ventrite I, with yellow-brown setae, and pectinate from antennomere III; processes of antennemeres III, IV, and V 1.1, 1.7, and 1.8 × as long as corresponding antennomeres; antennomere I robust; antennomere II shortest; antennomere III 1.7 × longer than II, as long as IV; antennomeres III–X with processes near apex, gradually lengthened apically; apical antennomere elongate, curved, 7.3 × longer than wide, and 1.9 × longer than previous one. **Pronotum** 1.3 × wider than long, subparallel-sided near base, gradually narrowed anteriorly from basal two-thirds; surface mostly with finer, sparser, and more regularly sized and spaced punctures than on head, larger and denser at sides; disc with a paired dimples near middle and a groove at midline, weakly swollen posteriorly; anterolateral carina short, almost one-third as long as pronotum; posterolateral carina almost reaching three-fifths length of pronotum; antescutellar area almost straight, weakly sinuate in dorsal view; pronotal posterior angles sharply projecting, slightly extended outward, and exceeding posterior edge of antescutellar area. **Scutellum** slightly raised; 1.2 × longer than wide, gradually narrowed posteriorly, and rounded at apex; surface coarse, densely pubescent. **Elytra** 2.7 × longer than combined width, parallel-sided, gradually narrowing near apices; disc striate, with irregularly sized and spaced punctures; interstriae weakly convex, with several large and deep punctures near apices; apices simply rounded. **Prosternum** with curved sides, slightly widened anteriorly, and anterior margin shallowly bisinuate; surface mostly with larger, sparser, and more regularly sized and spaced punctures than on head, especially at center; prosternal process robust, gradually tapered and curved dorsally at posterior end; hypomeron with rough surface, with larger, denser punctures than on prosternum; surface rugose at coxal cavities; notosternal antennal grooves (Fig. 2I) slightly widened posteriorly, with outer marginal carina, with several irregularly sized and spaced punctures posteriorly, glossy, and with pits. **Mesoventrite** with coarse surface, with shallow punctures; mesopleuron with rough surface, especially anteriorly. **Metaventrite** mostly with finer and denser punctures than on prosternum; disc with a groove at midline, not reaching anterior edge; metepisternum (Fig. 2J) narrow, gradually widened posteriorly, its greatest width as wide as outer edge of metacoxal plate; metacoxal plate (Fig. 2K) expanded inward, medially 1.6 × wider than laterally. **Legs** (Fig. 2O) slender; metatarsomere I 1.5 × longer than II–IV combined; metatarsomere II 1.3 × longer than III; metatarsomere V 1.2 × longer than II; claws simple. **Abdomen** with finer and denser punctures than on metaventrite; ventrite V narrowly rounded at posterior edge (Fig. 2L). **Aedeagus** (Fig. 2M–N) 4.3 × longer than wide; median lobe curved ventrally near apex, broadly bifurcate at apex with setae; lateral lobes slender with apical tooth inward, truncate and with long setae at apex; ventral lobe shorter and broader than median lobe, almost truncate at apical edge; phallobase globose basally with concave sides near apex, 1.5 × longer than wide, one-third as long as entire aedeagus. **Allotype female** (Fig. 2B) like male, except for following characters: 6.1 mm long and 1.8 mm wide; frontoclypeal region with anterior edge, 3.7 × wider than distance between antennal sockets (Fig. 2H); antennae (Fig. 2F) serrate, not exceeding metacoxal plate; antennomere III subrectangular, approximately twice longer than wide, 1.7 × longer than II, and 1.3 × longer than IV; apical antennomere 5.3 × longer than wide, and 1.8 × longer than X.

####### Type meterial.

**Holotype: Korea**: 1♂, Jeju Is., Hwasun gotjawal, Hwasun-ri, Andeok-myeon, Seogwipo-si, N33°15'52.88", E126°19'53.59", 120m alt., 12 May, 2016, J. B. Seung leg. (SNU). **Allotype: Korea**: 1♀, Jeju Is., Gyorae gotjawal, Gyorae-ri, Jocheon-eup, Jeju-si, N33°26'21.15", E126°40'12.75", 428m alt., flight intercept trap, 12 May–10 June, 2016, Seung and Jung leg. (SNU). **Paratypes: Korea**: 1♀, Jeju Is., Donnaeko, Sanghyo-dong, Seogwipo-si, N33°18'1.34", E126°34'49.02", 280m alt., 12 May, 2016, J. B. Seung leg. (SNU); 1♀, Jeju Is., Gyorae gotjawal, Gyorae-ri, Jocheon-eup, Jeju-si, N33°26'21.15", E126°40'12.75", 428m alt., flight intercept trap, 12 May–10 June, 2016, Seung and Jung leg. (SNU); 2♂1♀, Jeju Is. Hwasun gotjawal, Hwasun-ri, Andeok-myeon, Seogwipo-si, N33°15'52.62", E126°19'52.43", 128m alt., flight intercept trap, 12 May–10 June, 2016, Seung and Jung leg. (SNU); 1♂2♀, Seongpanak, Gyorae-ri, Jocheon-eup, Jeju-si, N33°23'10.82", E126°37'13.77", 752m alt., flight intercept trap, 12 May–10 June, 2016, Seung and Jung leg. (SNU).

####### Distribution.

Korea (Jeju Island).

####### Remarks.

*Microrhagusjejuensis* sp. n. similar to *M.foveolatus*, but is distinguished from *M.foveolatus* by following characters: frons with a groove at midline; pronotum with a short groove at midline; elytra elongate, width 2.7 × longer than combined width; lateral lobes of aedeagus short and truncate at apex. The structure of aedeagus resembles that of *M.pectinicornis*, but the latter species differs from the new species in longer processes of antennomeres (processes of antennomeres III, IV, and V 2.2, 3.6, and 4 × as long as corresponding antennomeres). Additionally, each process of male antennomeres III and IV is near base in *M.pectinicornis*, and not in *M.jejuensis*.

####### Etymology.

The species is named refers to its occurrence locality, Jeju Island.

###### Microrhagus mystagogus

Taxon classificationAnimaliaColeopteraEucnemidae

(Fleutiaux, 1923)

Fig. 3

Dirhagus mystagogus Fleutiaux, 1923: 309.

####### Diagnosis.

Body: mostly dull black. Head: antennae pectinate from antennomere IV in male. Prothorax: pronotum convex, as long as wide; notosternal antennal grooves subparallel-sided. Pterothorax: elytra 2.3 × longer than combined width; metepisternum narrow, slightly widened posteriorly, its greatest width narrower than outer edge of metacoxal plate; metacoxal plate expanded inward; abdominal ventrite V simply rounded at apex.

####### Redescription.

**Figure 3.**
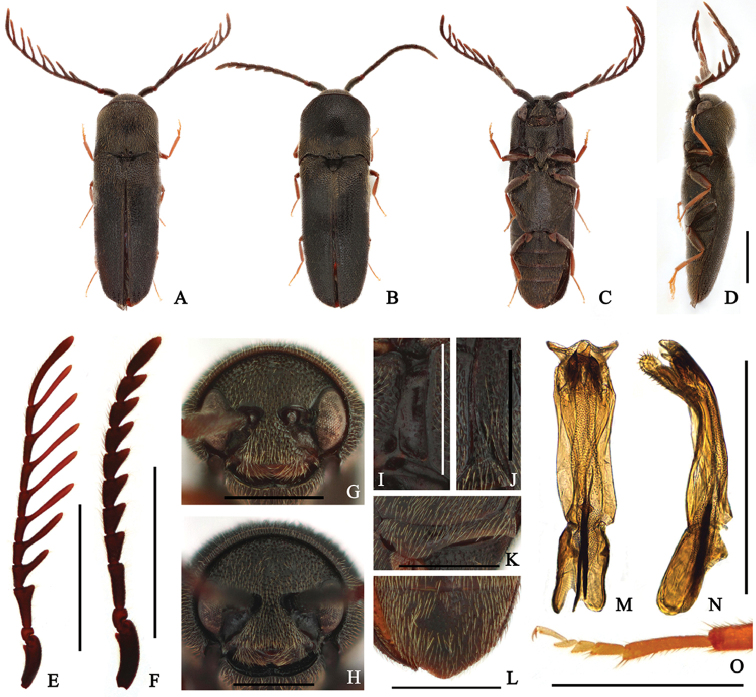
*Microrhagusmystagogus* (Fleutiaux, 1923). **A, C–E, G, I–O** male **B, F, H**, female **A, B** dorsal habitus **C** ventral habitus **D** lateral habitus **E–F** antenna **G–H** frons **I** antennal groove **J** metepisternum **K** metacoxal plate **L** abdominal ventrite V **M–N** aedeagus **O** metatarsus. Scale bar: 1 mm (**A–F**); 0.5 mm (**G–O**).

**Male** (Fig. 3A, C–D) 3.2–3.9 mm long and 1.0–1.2 mm wide. **Body** mostly black; antennae and femur dark red-brown; tibiae and tarsi yellow-brown; surface weakly glossy, with yellow pubescence. **Head** with circular punctures, becoming denser and irregularly sized near frontoclypeal region; frons without carina or groove at midline; frontoclypeal region slightly depressed at base, rounded and weakly sinuate at anterior edge, anterior edge 3.9 × wider than distance between antennal sockets (Fig. 3G). **Antennae** (Fig. 3E) almost exceeding metacoxal plate, with yellow-brown pubescence, and pectinate from antennomere IV; processes of antennomeres IV, V, and VI 1.4, 2.3 and 2.6 × as long as corresponding antennomeres; antennomere I stout; antennomere II shortest; antennomere III gradually expanded toward apex, 1.9 × longer than wide, three × longer than II, and 1.8 × longer than IV; antennomeres IV–X with processes near apex, gradually lengthened toward apex; apical antennomere strongly elongate, curved, 11.5 × longer than wide, and 2.9 × longer than previous one. **Pronotum** as long as wide, parallel-sided; surface with more regularly sized and spaced punctures than on head, slightly larger and sparser posteriorly; disc with a short carina at base of midline; anterolateral carina short, almost reaching one-third length of pronotum; posterolateral carina exceeding two-thirds length of pronotum; antescutellar area almost straight; pronotal posterior angles sharply projecting, exceeding posterior edge of antescutellar area. **Scutellum** subtriangular, 1.1 × wider than long, gradually narrowed posteriorly, and rounded at apex; surface rough, barely pubescent. **Elytra** 2.3 × longer than combined width, parallel-sided, gradually narrowing near apices; disc barely striate, with shallow and irregularly sized and spaced punctures; several large and deep punctures present near apices; apices simply rounded. **Prosternum** subparallel-sided, anterior margin shallowly bisinuate; surface mostly with punctures as on pronotum, slightly larger laterally; prosternal process robust basally, abruptly tapered and curved dorsally at posterior end; hypomeron mostly punctate as prosternum; surface wrinkled at coxal cavities; notosternal antennal grooves (Fig. 3I) subparallel-sided, with outer marginal carina, rarely punctate, glabrous, and with pits. **Mesoventrite** with coarse surface; mesopleuron with irregularly sized and spaced punctures, especially anteriorly. **Metaventrite** mostly with finer punctures than on prosternum, slightly larger laterally; disc with a groove at midline, not reaching anterior margin; metepisternum (Fig. 3J) widened posteriorly, its greatest width four-fifths of outer edge of metacoxal plate; metacoxal plate (Fig. 3K) expanded inward, medially 1.8 × wider than laterally. **Legs** (Fig. 3O) slender; metatarsomere I 1.3 × longer than II–IV combined; metatarsomere II 1.3 × longer than III; metatarsomere V 1.2 × longer than II; claws simple. **Abdomen** with finer and denser punctures than on metaventrite; ventrite V simply rounded at apex (Fig. 3L). **Aedeagus** (Fig. 3M–N) 3.9 × longer than wide, compressed dorsoventrally; median lobe slightly bent ventrally, fused with lateral lobes; lateral lobes strongly curved ventrally, enlarged near apex, densely setose; ventral lobe bifurcate apically, with dense short pubescence; phallobase strongly emarginate basally, 1.6 × longer than wide, approximately one-fifth as long as aedeagus. **Female** (Fig. 3B) is distinguished from male by following characters: body stouter, 3.4–3.9 mm long and 1.1–1.3 mm wide; frontoclypeal region (Fig. 3H) with anterior edge, 3.7 × wider than distance between antennal sockets; antennae (Fig. 3F) serrate, almost reaching metacoxal plate; antennomere III subrectangular, 3.1 × longer than wide, 2.3 × longer than II, and 1.6 × longer than IV; antennomeres IV–X gradually more strongly serrate; apical antennomere 3.5 × longer than wide, and approximately twice longer than X.

####### Specimens examined.

**<Gyeonggi-do>** 1♀, Deoksu-ri, Danwol-myeon, Yangpyeong-gun, N37°33'22.97", E127°41'13.65", 166m alt., flight intercept trap, 29 June–16 July, 2016, Seung and Jung leg. (SNU). **<Gangwon-do>** 1♀, Yeongheung-ri, Yeongwol-eup, Yeongwol-gun, N37°12'19.84", E128°27'17.77", 308m alt., flight intercept trap, 19 June–02 July, 2015, Seung and Lee leg. (SNU); 1♂, Beopheung-ri, Suju-myeon, Yeongwol-gun, N37°22'41.19", E128°15'15.50", 550m alt., flight intercept trap, 03–16 July. 2015, Seung and Lee leg. (SNU); 1♂2♀, Beopheung-ri, Suju-myeon, Yeongwol-gun, N37°22'41.19", E128°15'15.50", 550m alt., flight intercept trap, 05–29 June, 2016, Seung and Jung leg. (SNU).

####### Distribution.

Korea (New record), Japan, Russia (Far East).

####### Remarks.

*Microrhagusmystagogus* is easily distinguished from other Korean *Microrhagus* species by its antennae: male antennae pectinate from antennomere IV. Also, convex pronotum and structure of aedeagus are characteristic. Its aedeagal structure is well-illustrated in Kovalev’s (2013) work.

###### Microrhagus ramosus

Taxon classificationAnimaliaColeopteraEucnemidae

Fleutiaux, 1902

Fig. 4

Microrhagus ramosus Fleutiaux, 1902: 24.

####### Diagnosis.

Body: mostly weakly shiny black. Head: frons with a weak carina at midline; antennae pectinate from antennomere III in male. Prothorax: pronotum with dense punctures, average distance between punctures smaller than puncture diameter, disc with paired dimples at middle; notosternal antennal grooves slightly expanded posteriorly. Pterothorax: elytra 2.2 × longer than combined width; metepisternum gradually widened posteriorly, its greatest width wider than outer edge of metacoxal plate; metacoxal plate expanded inward. Abdomen: abdominal ventrite V weakly narrowly rounded at apex.

####### Redescription.

**Figure 4.**
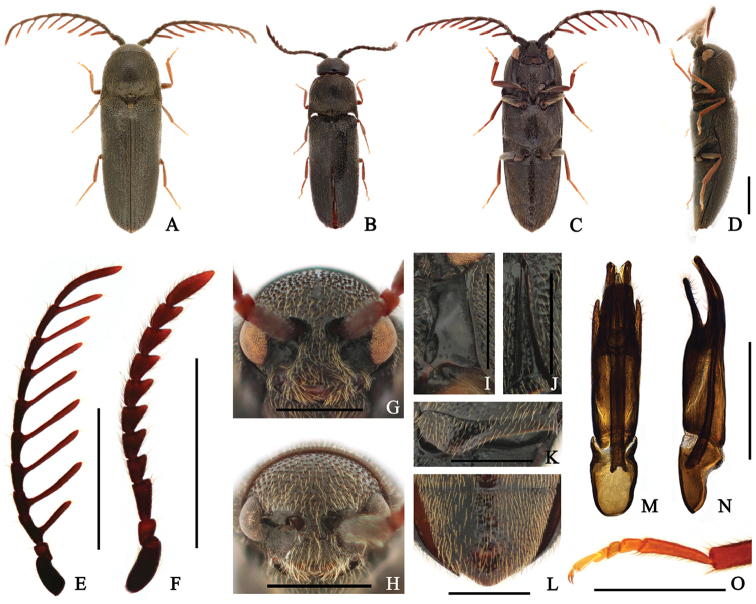
*Microrhagusramosus* (Fleutiaux, 1902). **A, C–E, G, I–O** male **B, F, H** female **A, B** dorsal habitus **C** ventral habitus **D** lateral habitus **E–F** antenna **G–H** frons **I** antennal groove **J** metepisternum **K** metacoxal plate **L** abdominal ventrite V **M–N** aedeagus **O** metatarsus. Scale bar: 1 mm (**A–F**); 0.5 mm (**G–O**).

**Male** (Fig. 4A, C, D) 3.3–4.8 mm long and 1.0–1.5 mm wide. **Body** mostly black; antennal branches and tibiae orange-brown; tarsi yellow-brown; surface weakly glossy, with yellow pubescence. **Head** regularly sized, circular punctures, becoming finer and denser near frontoclypeal region; frons with a weak carina at midline; frontoclypeal region slightly depressed at base, weakly sinuate at anterior edge, anterior edge 4.2 × wider than distance between antennal sockets (Fig. 4G). **Antennae** (Fig. 4E) almost exceeding metacoxal plate, with yellow-brown pubescence, pectinate from antennomere III; processes of antennomeres III, IV, and V 1.4, 2.5, and 2.4 × as long as corresponding antennomeres; antennomere I robust; antennomere II shortest; antennomere III with process near base, 1.7 × longer than II, and 1.3 × longer than IV; antennomere IV with process at mid-length; antennomeres V–X with processes near apex, gradually lengthened and narrowing toward apex; apical antennomere strongly elongate, curved, 9.4 × longer than wide, and 2.2 × longer than X. **Pronotum** 1.1 × wider than long, subparallel-sided near base, gradually narrowed anteriorly from basal two-thirds; surface mostly with denser punctures than on head, average distance between punctures smaller than puncture diameter; disc with paired dimples at middle and a short carina at base of midline, and symmetrically depressed near base; anterolateral carina almost reaching pronotal mid-length; posterolateral carina almost exceeding pronotal mid-length, fused with anterolateral carina in some; antescutellar area weakly notched in dorsal view; pronotal posterior angles sharply projecting, exceeding posterior edge of antescutellar area. **Scutellum** raised; triangular, 1.3 × longer than wide, gradually narrowed posteriorly, and rounded at apex; surface rough, densely pubescent, especially near apex. **Elytra** 2.2 × longer than combined width, subparallel-sided, gradually narrowed posteriorly; disc weakly striate, with irregularly sized and spaced punctures; interstriae slightly convex, with several large and deep punctures near apices; apices simply rounded. **Prosternum** with curved sides, anterior margin slightly bisinuate; surface with more scattered and regularly sized punctures than on pronotum; prosternal process stout, gradually tapered and curved dorsally at posterior end; hypomeron with denser, and larger punctures than on prosternum; notosternal antennal grooves (Fig. 4I) slightly widened posteriorly, barely punctate, glabrous, and with pits. **Mesoventrite** with coarse surface, with irregularly sized and spaced punctures; mesopleuron with rough surface, especially anteriorly. **Metaventrite** with finer and sparser punctures than on prosternum, especially at middle; disc with a weak groove at midline, not reaching anterior margin; metepisternum (Fig. 4J) gradually widened posteriorly, its greatest width 1.2 × wider than outer edge of metacoxal plate; metacoxal plate (Fig. 4K) expanded inward, medially 2.3 × wider than laterally. **Legs** (Fig. 4O) slender; metatarsomere I 1.3 × longer than II–IV combined; metatarsomere II 1.3 × longer than III; metatarsomere V 1.5 × longer than II; claws simple. **Abdomen** punctate as metaventrite; ventrite V narrowly rounded at apex (Fig. 4L). **Aedeagus** (Fig. 4M–N) five × longer than wide; median lobe slightly curved ventrally, deeply bifurcate at apex; lateral lobes as long as median lobe, subparallel-sided, curved ventrally, with basally attached secondary lateral lobes; secondary lateral lobes parallel-sided, curved ventrally, pointed at apex; ventral lobe shorter than median lobe, parallel-sided, truncate at apex, and densely pubescent; phallobase rectangular, 1.6 × longer than wide, almost one-third of entire length of aedeagus. **Female** (Fig. 4B) is distinguished from male by following characters: frontoclypeal region with anterior edge, four × longer than distance between antennal sockets (Fig. 4H); antennae (Fig. 4F) serrate, not reaching metacoxal plate; antennomere I stout; antennomere II short, as long as IV; antennomere III 1.9 × longer than wide, 1.7 × longer than IV; antennomere IV–X gradually lengthened, narrowing and more strongly toothed toward antennal apex; apical antennomere elongate, 3.5 × longer than wide, 2.2 × longer than previous one.

####### Specimens examined.

**<Gangwon-do>** 3♂1♀, Beopheung-ri, Suju-myeon, Yeongwol-gun, N37°22'41.19", E128°15'15.50", 550m alt., flight intercept trap, 19 June–02 July, 2015, Seung and Lee leg. (SNU); 2♂1♀, Deokgu-ri, Sangdong-eup, Yeongwol-gun, N37°5'34.46", E128°48'59.53", 648m alt., flight intercept trap, 19 June–02 July 2015, Seung and Lee leg. (SNU); 3♂, Deokgu-ri, Sangdong-eup, Yeongwol-gun, N37°5'34.46", E128°48'59.53", 648m alt., flight intercept trap, 02–16 July, 2015, Seung and Lee leg. (SNU); 5♂, Hoenggye-ri, Daegwanryeong-myeon, Pyeongchang-gun, N37°40'58.95", E128°45'21.80", 830m alt., flight intercept trap, 05–29 June, 2016, Seung and Jung leg. (SNU); 1♂, Jindong-ri, Girin-myeon, Inje-gun, N37°58'11.10", E128°24'23.97", 619m alt., 13 July, 2016, M. S. Oh leg. (SNU). **<Jeollanam-do>** 1♂, Donggok-ri, Ongnyong-myeon, Gwangyang-si, N35°5'13.37", E127°36'48.93", 577m alt., flight intercept trap, 04–15 July, 2016, Seung and Lee leg. (SNU). **<Jeju Is.>** 1♂, Gyorae gotjawal, Gyorae-ri, Jocheon-eup, Jeju-si, N33°26'21.15", E126°40'12.75", 428m alt., flight intercept trap, 13 May–10 June, 2016, Seung and Jung leg. (SNU); 1♂, Seongpanak, Gyorae-ri, Jocheon-eup, Jeju-si, N33°23'10.82", E126°37'13.77", 752m alt., flight intercept trap, 13 May–10 June, 2016, Seung and Jung leg. (SNU).

####### Distribution.

Korea, Japan.

####### Remarks.

*Microrhagusramosus* shows morphological variation as below: pronotal anterolateral carina and posterolateral carina obscure in some, appearing fused in some; ventral lobe of aedeagus sub-parallel sided in some, slightly widened near apex.

## Supplementary Material

XML Treatment for Microrhagus

XML Treatment for Microrhagus foveolatus

XML Treatment for Microrhagus jejuensis

XML Treatment for Microrhagus mystagogus

XML Treatment for Microrhagus ramosus
